# Medullary Thyroid Carcinoma With Exon 2 p.L56M RET Variant: Clinical Particular Features in Two Patients

**DOI:** 10.3389/fendo.2018.00398

**Published:** 2018-07-19

**Authors:** Rosa M. Paragliola, Rosa M. Lovicu, Giampaolo Papi, Ettore Capoluongo, Angelo Minucci, Giulia Canu, Alfredo Pontecorvi, Salvatore M. Corsello

**Affiliations:** ^1^Unit of Endocrinology, Università Cattolica del Sacro Cuore, Rome, Italy; ^2^Unit of Biochemistry and Clinical Biochemistry, F. Policlinico Gemelli IRCCS, Università Cattolica del Sacro Cuore, Rome, Italy

**Keywords:** medullary thyroid carcinoma, MEN2, RET, pheochromocytoma, L56M

## Abstract

RET (REarranged during Transfection) proto-oncogene variants are essential for the development of familial and sporadic forms of medullary thyroid carcinoma (MTC). The most frequent variants are usually located in exons 10, 11, and 13 through 16 of the RET gene. We report two cases of apparently sporadic MTC associated with the variant in exon 2 of *RET* gene. Patient 1, a 62-year old man who had undergone adrenalectomy for a 5 cm pheochromocytoma, was screened for type 2 multiple endocrine neoplasia (MEN 2) which showed elevated basal and post-intravenous calcium gluconate calcitonin levels. A fine needle aspiration biopsy (FNAB) confirmed the suspicion of MTC. The patient underwent total thyroidectomy and lymphadenectomy, and the histology showed C-cell hyperplasia with medullary microcarcinoma. Patient 2, a 57 years old woman, underwent total thyroidectomy for toxic multinodular goiter. Pre-operative FNAB had shown benign features, while basal calcitonin levels were only borderline increased. Final histology revealed medullary multifocal microcarcinoma. Genetic testing for RET protoncogene on DNA extracted from peripheral blood was performed in both patients and a missense variant on exon 2 (c.166C>A, p.L56M) was identified. To our knowledge, these are the first time two cases of MTC associated to RET p.L56M variant. Interestingly, one patient had also a pheochromocytoma suggesting a possible pathogenetic role of this variant in the genesis of MEN2A. While the association of this variant with MTC or MEN2A has been never reported, it has been described in association with Hirschsprung's disease.

## Background

Medullary thyroid carcinoma (MTC), originating from parafollicular C-cells, accounts for 5% of all thyroid cancers and can occur as sporadic (75% of cases) or hereditary (25%) disease (1). Germline *RET* proto-oncogene variants play a crucial pathogenetic role and are found in the majority of hereditary forms (98%). In fact, only few families with hereditary MTC do not show any germline variant (2). On the other hand, somatic *RET* variants are responsible for approximately 40% of cases of sporadic MTC, according to data of COSMIC database published in 2015 (3). The *RET* proto-oncogene, located on chromosome 10, codifies for a member of the tyrosine-kinase family receptors which is expressed on C cells, parathyroid glands, adrenal medulla and urogenital tract. In the hereditary forms, the causative role of the germline *RET* variants has been clearly demonstrated, and there appears to be a strict correlation between genotype and phenotype, leading to different degree of risk related to the aggressiveness of the tumor and the clinical syndrome [familiar medullary thyroid cancer (FMTC), and type 2A and type 2B multiple endocrine neoplasia (MEN) (4)]. The “classic MEN2A” phenotype is characterized by the concomitant occurrence of pheochromocytoma and primary hyperparathyroidism. In addition to the “classic MEN2A,” other forms associated with cutaneous lichen amyloidosis (5) and with Hirschsprung disease have been reported (6). MEN2A is primarily associated with variants in the *RET* gene causing substitution of cysteines at codons 609, 611, 618, and 620 in exon 10, and particularly with the Cys634Arg alteration in exon 11 (3).

On the contrary, MEN2B, where MTC which is associated with pheochromocytoma and other additional clinical features (e.g., mucosal neuromas), is mostly associated with the Met918Thr variant in exon 16.

While genetic testing is mandatory whenever the familial form of MTC is suspected, germline *RET* testing may also be recommended in all patients with newly diagnosed C cell hyperplasia (CCH) or apparently sporadic MTC, since approximately 7% of patients, who would appear to have a “sporadic” form, in truth have an unsuspected germline variant in the *RET* proto-oncogene.

In the past decade, additional *RET* variants, unknown at the time of the International *RET* Consortium (7), have been discovered. These include very rare germline *RET* variants (8) often observed in a single affected member. However, the germline *RET* proto-oncogene variants identified during the past 20 years are localized in specific regions and involve eight exons (exons 5, 8, 10, 11, 13, 14, 15, 16) (9).

We here report the first two cases of MTC associated with the variant of exon 2 of *RET* proto-oncogene causing the substitution of Leucine with Methionine at codon 56 (p.L56M; rs145633958), which, to the best of our knowledge, has not previously been reported in association to MTC. In one case, the diagnosis of MEN2A cannot be excluded, considering the concomitant occurrence of pheochromocytoma.

Interestingly, the above-mentioned variant has been described in association with Hirschsprung disease, which has been excluded in these two patients.

## Case presentation and description of laboratory investigations and diagnostic tests

Patient 1: A 62-year man came to our attention for an incidentally discovered right adrenal mass of 5 cm in diameter, detected during abdominal ultrasound evaluation for suspected nephrolithiasis. An abdominal computer tomography scan showed an adrenal mass suggestive of adrenal adenoma, without the typical radiological characteristics of pheochromocytoma.

The patient had hypertension which was well controlled by lercanidipine 10 mg/day. His family history was negative for endocrine disease.

Blood cortisol, aldosterone, adrenal androgens levels, and 24-h urinary catecholamine and metanephrines levels were normal. However, in consideration of the size of the tumor, the patient was referred for laparoscopic adrenalectomy. Surprisingly, final histology revealed a 55 mm pheochromocytoma. Because of the presence of bilateral thyroid micronodules (7 mm on right lobe and 9 mm on left lobe), serum calcitonin was measured, showing mildly elevated basal levels (20 pg/ml) and post i.v. calcium gluconate levels (296 and 366 pg/ml 2 and 3 min after the infusion, respectively). Fine needle aspiration biopsy (FNAB) performed on the left nodule confirmed the suspicion of MTC. The patient underwent total thyroidectomy and central neck dissection. Final pathology showed diffuse CCH and a 4 mm medullary microcarcinoma on the left nodule which previously had undergone FNAB, and no lymph node involvement.

Patient 2: A 58 years-old woman came to our attention for tachycardia. Laboratory evaluation showed mild hyperthyroidism and thyroid ultrasound revealed a multinodular goiter. Thyroid scintiscan confirmed the diagnosis of a toxic multinodular goiter, with a 2 cm hyperfunctioning left nodule. A FNAB evaluation performed on the most relevant non-hyperfunctioning nodule (15 mm on right lobe) showed was benign features (Thy 2). Serum calcitonin was borderline increased (13.2 pg/ml). Due to the symptoms of hyperthyroidism and dysphagia, the patient underwent total thyroidectomy and the final histology showed struma with CCH and medullary multifocal microcarcinoma (maximum diameter 6 mm on left lobe).

### Molecular analysis of *RET*-protoncogene

After obtaining informed consent, DNA was isolated from peripheral blood by a manual method (Roche Diagnostics, Basel, Switzerland, http://www.roche.com/index.htm). Molecular analysis of the *RET* onco-gene was performed by sequencing of the coding region and exon-intron boundaries of the exons 2, 5, 8, 10, 11, 13–16, as routinely performed in our laboratory. Primers used and the respective annealing temperatures are reported in Table 1. PCR products were sequenced using the Big Dye Terminator v3.1 Cycle Sequencing kit (Applied Biosystems, Foster City, CA, USA, http://www.appliedbiosystems.com/absite/us/en/home.html) in an automated sequencer ABI Prism 3500 Genetic Analyzer (Applied Biosystems).

**Table 1:** Primers used for *RET* amplification and sequencing.

**Oligo name**	**Sequence (5′ 3′)**	**Annealing temperature**
		**(Ta) [°C]**
RET-2F	GCT TCC CCT GTT TCC TTT TC	
RET-2R	AGT GTC AGC GGC TGT GAT AA	
RET-5F	CGT GCA GCA TTC TAA GGT CTC	
RET-5R	CAT GTG TGT AGG GTG CTG CT	
RET-8F	TGC TCC TGG CAC TGT CTT	
RET-8R	TGG GGA CCA ATC ACT GTA CTC	
RET-1OF	GGA CAC TGC CCT GGA AAT A	
RET-1OR	ACT CGC CTC CCA GCA ATT T	
RET-11F	ATA CGC AGC CTG TAC CCA CT	
		58
RET-11R	AAA TGG GGG CAG AAC ACA	
RET-13F	CCA TCC TGA CCT GGT ATG GT	
RET-13R	AAA CAG GGC AGG AGC AGT AG	
RET-14F	GTC CAC CCC CTT ACT CAT TG	
RET-14R	GTG GTG AGC CAT AGC ATG G	
RET-15F	CCC CCG GCC CAG GTC TC	
RET-15R	GCT CCA CTA ATC TTC GGT ATC TTT	
RET-16F	TCT CCT TTA CCC CTC CTT CC	
RET-16R	CAG TGA GGG GGT CAT TGC	

*Sanger* sequencing revealed in both patients a missense variant on exon 2 (c.166C>A, p.L56M). This change from cytosine to adenine causes a substitution of Leucine with Methionine at codon 56. Genetic testing was performed also in first degree relatives and the same variant was found in the son of Patient 1, who is being followed with periodic ultrasonography and calcitonin measurements. In order to assess the frequency of this variant within our patient population, we also screened 100 healthy controls (200 alleles) by high-resolution melting analysis; these additional samples were used since the p.L56M was never found within the previous 250 patients referred to our laboratory with the suspicion of MEN2 or MTC. All screened subjects were from Northern, Central and Southern Italy. This screening resulted mute for the above-mentioned variant, indicating a frequency of <1%.

## Discussion

The *RET* proto-oncogene, located on the long arm of chromosome 10 (10q11.2), was first identified in 1985 by transfection of NIH 3T3 cells with human lymphoma DNA (10). The protein encoded by *RET* is a cellular tyrosine kinase transmembrane receptor that is divided into three domains: an N-terminal extracellular domain with cadherin-like regions, a cysteine-rich transmembrane domain, and a cytoplasmic domain with tyrosine kinase activity (11). The activation of *RET* stimulates multiple pathways promoting cell growth, proliferation, differentiation and survival (4).

Once the role of the mutated *RET* in the development of hereditary forms of MTC became clear, *RET* genetic screening was introduced into clinical practice (8), providing an important contribution in the diagnosis of MTC. In fact, before the introduction of the *RET* genetic screening test in clinical practice, the diagnosis of MTC was exclusively relying on FNAB and serum calcitonin measurements (12), which made the identification of making the identification of the familial forms quite challenging. Nowadays, genetic screening is aimed at the early identification of family members who carry the same mutation as the index case and to propose an early treatment considering of the degree of risk associated with the detected variant (13).

Furthermore, the evidence that germline *RET* variants are present in about 6–7% of “apparently sporadic” cases of MTC confirms the need to perform genetic screening in all patients with MTC (8). If a variant is found, the case is then reclassified as hereditary, and genetic screening of the first-degree relatives is strongly recommended (9). Following such recommendation, we performed genetic testing in our two patients following the diagnosis of “sporadic” MTC and, for the first time, a variant in exon 2 of RET was detected in association with MTC. Moreover, in Patient 1 the diagnosis of MEN2A cannot be ruled out, in consideration of the concomitant occurrence of MTC and pheochromocytoma.

As often reported for the “new” variants, the question is if the *RET* germline variant represents a driving force of MTC or if it is an incidental finding due to the increased use of screening (14). In literature, rare variants which cannot be considered to be “polymorphisms” and which have an uncertain role in the pathogenesis on MTC are described as variants of unknown significance (VUS) (15). In our case, the frequency of p.L56M variant in the general population is <1%, and cannot be considered a polymorphisms. Recently, the ClinVar database identified this mutation in the MEN2A. The clinical significance as been defined as “benign/likely benign,” because “it is a conservative change, it occurs at a poorly conserved position in the protein, it is predicted to be benign by multiple in silico algorithms, and/or has population frequency not consistent with disease”^1^. On the other hand, further studies are necessary to confirm the possible pathogenic role of this variant in MTC development: in fact, in clinical practice, genetic studies are often limited to exons 5, 8, 10, 11, 13, 14, 15, 16, where the majority of known *RET* pathogenic germline variant is localized. Whether the variant described by our group can be considered “pathogenic” in MTC or even in MEN2A is a hypothesis that needs to be confirmed. However, an interesting consideration is that this very same variant had previously been reported in association with Hirschsprung's disease, the congenital absence of ganglion cells in the submucosal and myenteric plexi of the gut. *RET* is the main gene implicated in this condition which, as mentioned earlier, represents a rare clinical feature of a MEN 2A clinical variant (6). Approximately 50% of familial cases of MTC and 7–35% of non-familial cases of MTC have loss-of-function germline RET variants (16). The concomitant occurrence of Hirschsprung's disease and MEN 2 is a relatively rare event, due to the presence of a “Janus” variant in the *RET* proto-oncogene: these variants can act both as a gain-of-function and a loss-of-function variant. To date, four missense exon 10 *RET* variants have been implicated in this association, most frequently in codon C620 (mostly C620R and occasionally C620S, and rarely C620W), but also in other areas (e.g., C609, C611, and C618) (6). Common variants in the *RET* promoter (rs10900296; rs10900297), at a SOX10 binding site in intron 1 (rs2435357), and in exon 2 (rs1800858; c.135G>A;p.A45A) have also been associated with Hirschsprung's disease, suggesting that common as well as rare variants might influence the occurrence of Hirschsprung's disease (17). It is important to mention that the presence of Hirschsprung's disease in our two patients was excluded based on clinical evaluation. However, the association between MTC and exon 2 p.L56M variant, which in turn is also related with a disease involved in MEN 2 phenotype, underlines the possibility of a pathogenic role of p.L56M variant in MTC. These findings need further confirmatory results. Nevertheless, it is important to underline the importance to expand the genetic evaluation for *RET* germline variants in MTC or MEN2A patients also to exon 2, especially if genetic testing has excluded the presence of the more common variants located within other exons.

## Author contributions

RP, RL, GP, EC, AM, GC, AP, and SC have equally made their contribution to the work and approved it for publication.

### Conflict of interest statement

The authors declare that the research was conducted in the absence of any commercial or financial relationships that could be construed as a potential conflict of interest.

## Footnotes

^1^https://preview.ncbi.nlm.nih.gov/clinvar/variation/36723/evidence/

## References

[1] Wells SA Jr Pacini F Robinson BG Santoro M. Multiple endocrine neoplasia type 2 and familial medullary thyroid carcinoma: an update. J Clin Endocrinol Metab. (2013) 98:3149–64. 10.1210/jc.2013-1204 23744408 PMC5399478

[2] Romei C Mariotti S Fugazzola L Taccaliti A Pacini F Opocher G. Multiple endocrine neoplasia type 2 syndromes (MEN 2): results from the ItaMEN network analysis on the prevalence of different genotypes and phenotypes. Eur J Endocrinol. (2010) 163:301–8. 10.1530/EJE-10-0333 20516206

[3] Romei C Ciampi R Elisei R. A comprehensive overview of the role of the RET proto-oncogene in thyroid carcinoma. Nat Rev Endocrinol. (2016) 12:192–202. 10.1038/nrendo.2016.11 26868437

[4] Torino F Paragliola RM Barnabei A Corsello SM. Medullary thyroid cancer: a promising model for targeted therapy. Curr Mol Med. (2010) 10:608–25. 20712590 10.2174/156652410792630607

[5] Scapineli JO Ceolin L Punales MK Dora JM Maia AL. MEN 2A-related cutaneous lichen amyloidosis: report of three kindred and systematic literature review of clinical, biochemical and molecular characteristics. Fam Cancer (2016) 15:625–33. 10.1007/s10689-016-9892-6 26920351

[6] Coyle D Friedmacher F Puri P. The association between Hirschsprung's disease and multiple endocrine neoplasia type 2a: a systematic review. Pediatr Surg Int. (2014) 30:751–6. 10.1007/s00383-014-3538-2 24972642

[7] Eng C Clayton D Schuffenecker I Lenoir G Cote G Gagel RF. The relationship between specific RET proto-oncogene mutations and disease phenotype in multiple endocrine neoplasia type 2. International RET mutation consortium analysis. JAMA (1996) 276:1575–9. 8918855

[8] Romei C Tacito A Molinaro E Agate L Bottici V Viola D. Twenty years of lesson learning: how does the RET genetic screening test impact the clinical management of medullary thyroid cancer? Clin Endocrinol. (2015) 82:892–9. 10.1111/cen.12686 25440022

[9] Elisei R Alevizaki M Conte-Devolx B Frank-Raue K Leite V Williams GR. 2012 European thyroid association guidelines for genetic testing and its clinical consequences in medullary thyroid cancer. Eur Thyroid J. (2013) 1:216–31. 10.1159/000346174 24783025 PMC3821492

[10] Takahashi M Ritz J Cooper GM. Activation of a novel human transforming gene, ret, by DNA rearrangement. Cell (1985) 42:581–8. 2992805 10.1016/0092-8674(85)90115-1

[11] Arighi E Borrello MG Sariola H. RET tyrosine kinase signaling in development and cancer. Cytokine Growth Factor Rev. (2005) 16:441–67. 10.1016/j.cytogfr.2005.05.010 15982921

[12] Elisei R Bottici V Luchetti F Di Coscio G Romei C Grasso L. Impact of routine measurement of serum calcitonin on the diagnosis and outcome of medullary thyroid cancer: experience in 10,864 patients with nodular thyroid disorders. J Clin Endocrinol Metab. (2004) 89:163–8. 10.1210/jc.2003-030550 14715844

[13] American Thyroid Association Guidelines Task Force Kloos RT Eng C Evans DB Francis GL Gagel RF. Medullary thyroid cancer: management guidelines of the American Thyroid Association. Thyroid (2009) 19:565–612. 10.1089/thy.2008.0403 19469690

[14] Orgiana G Pinna G Camedda A De Falco V Santoro M Melillo RM. A new germline RET mutation apparently devoid of transforming activity serendipitously discovered in a patient with atrophic autoimmune thyroiditis and primary ovarian failure. J Clin Endocrinol Metab. (2004) 89:4810–6. 10.1210/jc.2004-0365 15472167

[15] Crockett DK Piccolo SR Ridge PG Margraf RL Lyon E Williams MS. Predicting phenotypic severity of uncertain gene variants in the RET proto-oncogene. PLoS ONE (2011) 6:e18380. 10.1371/journal.pone.0018380 21479187 PMC3068179

[16] Attie T Pelet A Edery P Eng C Mulligan LM Amiel J. Diversity of RET proto-oncogene mutations in familial and sporadic Hirschsprung disease. Hum Mol Genet. (1995) 4:1381–6. 7581377 10.1093/hmg/4.8.1381

[17] Emison ES Garcia-Barcelo M Grice EA Lantieri F Amiel J Burzynski G. Differential contributions of rare and common, coding and noncoding Ret mutations to multifactorial Hirschsprung disease liability. Am J Hum Genet. (2010) 87:60–74. 10.1016/j.ajhg.2010.06.007 20598273 PMC2896767

